# Supplementary material for: Wolbachia Infections in Aedes aegypti Differ Markedly in Their Response to Cyclical Heat Stress
Source: PLoS Pathog. 2017 Jan 5;13(1):e1006006. doi: 10.1371/journal.ppat.1006006 (PMC5215852; doi:10.1371/journal.ppat.1006006)
Supplement: S1 Fig — Incubators were set to a constant 26°C or a cycling 26–32°C, 26–34.5°C or 26–37°C. Temperature was measured by submerging data loggers in plastic trays filled with 500 mL water, identical to the trays used for rearing larvae. Data shown were averaged from seven days of measurements; error bars represent standard deviations. (PDF) [file ppat.1006006.s001.pdf]

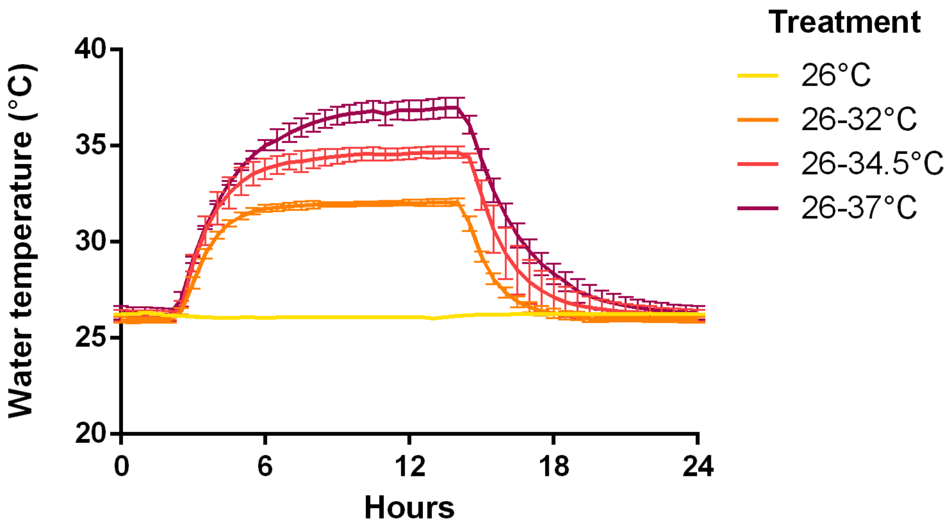

Figure S1. Diurnal temperature fluctuations in incubators set to a constant 26°C or a cycling 26-32°C, 26-34.5°C or 26-37°C. Temperature was measured by submerging data loggers in plastic trays filled with 500 mL water, identical to the trays used for rearing larvae. Data shown were averaged from seven days of measurements; error bars represent standard deviations.
