# Supplementary material for: Wolbachia Infections in Aedes aegypti Differ Markedly in Their Response to Cyclical Heat Stress
Source: PLoS Pathog. 2017 Jan 5;13(1):e1006006. doi: 10.1371/journal.ppat.1006006 (PMC5215852; doi:10.1371/journal.ppat.1006006)
Supplement: S2 Fig — Wing length of (A) females and (B) males reared at a constant 26°C, cycling 26–32°C and cycling 26–37°C. Thirty adults were measured per treatment. Data were normally distributed according to Shapiro-Wilk tests. Analysis of variance finds a significant effect of temperature regime on wing length for both females (one-way ANOVA: F2,356 = 11.203, P < 0.0001) and males (F2,357 = 9.381, P = 0.0001) but no effect of infection type for either sex (females: F3,355 = 0.313, P = 0.816, males: F3,356 = 1.714, P = 0.164). Increasing maximum temperatures had a negative effect on wing length for all infection types and both sexes, with the 26–37°C regime being the most stressful. (PDF) [file ppat.1006006.s002.pdf]

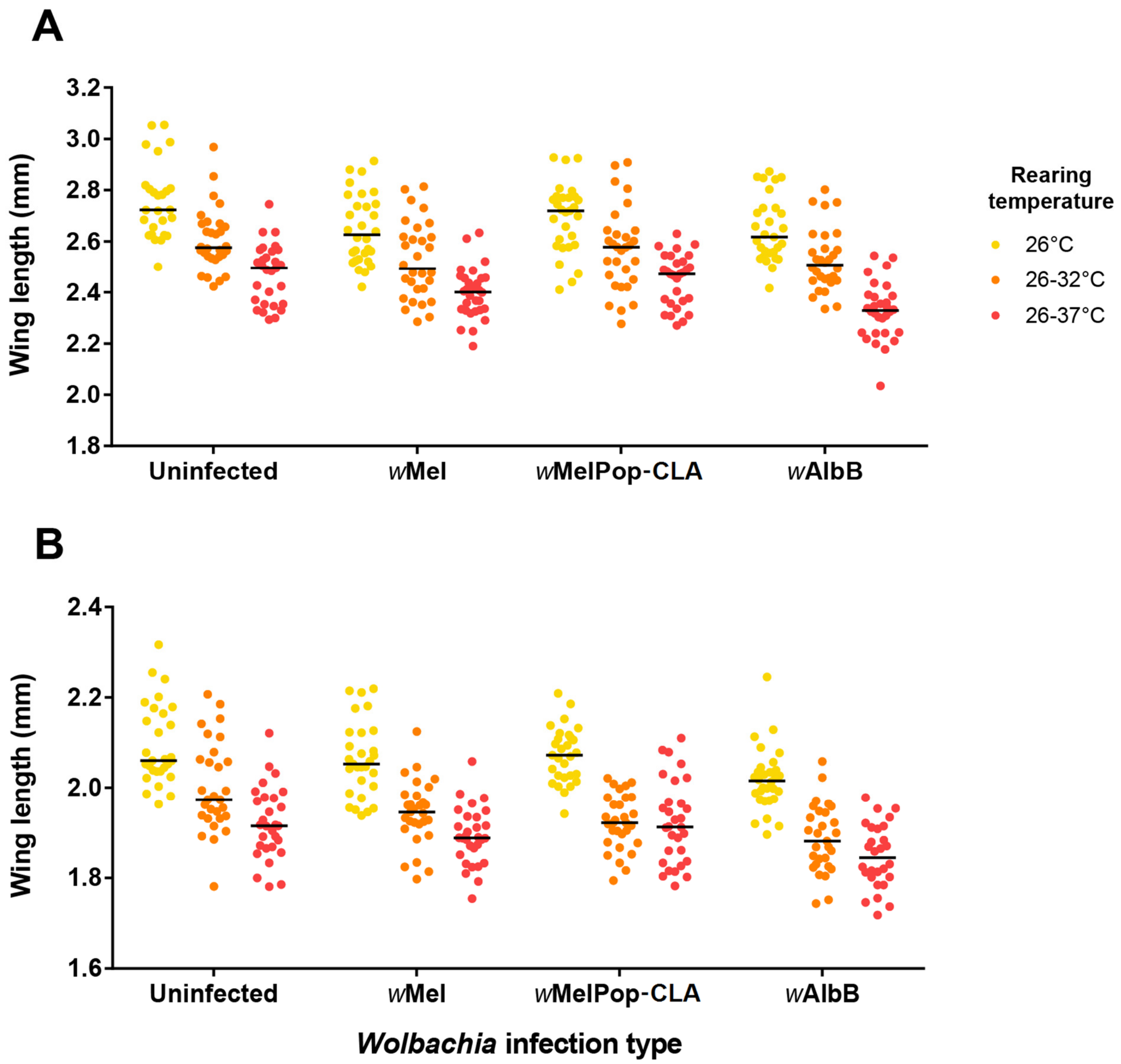

Figure S2. Wing length of (A) females and (B) males reared at a constant 26°C, cycling 26-32°C and cycling 26-37°C. Data were normally distributed according to Shapiro-Wilk tests. Analysis of variance finds a significant effect of temperature regime on wing length for both females (one-way ANOVA: $F_{2,356} = 11.203$, $P < 0.0001$) and males ($F_{2,357} = 9.381$, $P = 0.0001$) but no effect of infection type for either sex (females: $F_{3,355} = 0.313$, $P = 0.816$, males: $F_{3,356} = 1.714$, $P = 0.164$). Increasing maximum temperatures had a negative effect on wing length for all infection types and both sexes, with the 26-37°C regime being the most stressful.
