# Supplementary material for: Wolbachia Infections in Aedes aegypti Differ Markedly in Their Response to Cyclical Heat Stress
Source: PLoS Pathog. 2017 Jan 5;13(1):e1006006. doi: 10.1371/journal.ppat.1006006 (PMC5215852; doi:10.1371/journal.ppat.1006006)
Supplement: S1 Table — (PDF) [file ppat.1006006.s003.pdf]

S1 Table. Primers used in qPCR.

Specificity	Name	Sequence (5'-3')	Reference
Aedes	mRpS6_F	AGTTGAACGTATCGTTTCCCGCTAC	(1)
	mRpS6_R	GAAGTGACGCAGCTTGTGGTCGTCC	
Aedes aegypti	aRpS6_F	ATCAAGAAGCGCCGTGTCG	(1)
	aRpS6_R	CAGGTGCAGGATCTTCATGTATTCG	
*w*Mel	w1_F	AAAATCTTTGTGAAGAGGTGATCTGC	(1)
	w1_R	GCACTGGGATGACAGGAAAAGG	
*w*MelPop-CLA	wMelpop_F	CTCATCTTTACCCCGTACTAAAATTTC	(2)
	wMelpop_R	TCTTCCTCATTAAGAACCTCTATCTTG	
*w*AlbB	wAlbB_F	CCTTACCTCCTGCACAACAA	(3)
	wAlbB_R	GGATTGTCCAGTGGCCTTA	

qPCR = quantitative real-time polymerase chain reaction.

S1 Table references

1. Lee SF, White VL, Weeks AR, Hoffmann AA, Endersby NM. High-throughput PCR assays to monitor *Wolbachia* infection in the dengue mosquito (*Aedes aegypti*) and *Drosophila simulans*. Applied and environmental microbiology. 2012;78(13):4740-3.

2. Ritchie SA, Townsend M, Paton CJ, Callahan AG, Hoffmann AA. Application of *w*MelPop *Wolbachia* strain to crash local populations of *Aedes aegypti*. PLoS neglected tropical diseases. 2015;9(7):e0003930.

3. Axford JK, Ross PA, Yeap HL, Callahan AG, Hoffmann AA. Fitness of *w*AlbB *Wolbachia* Infection in *Aedes aegypti*: Parameter Estimates in an Outcrossed Background and Potential for Population Invasion. The American journal of tropical medicine and hygiene. 2016;94(3):507-16.
